# Fatal gunshot trauma of a child: A case from colonial Cyprus

**DOI:** 10.1177/00258024231202563

**Published:** 2023-09-24

**Authors:** Constantine Eliopoulos, Konstantinos Moraitis, Matteo Borrini, Joel Irish, Xenophon Kallis, Panayiotis Manoli, Marios Chimonas, Marios Cariolou

**Affiliations:** 1 School of Biological and Environmental Sciences, 4589 Liverpool John Moores University, Liverpool, UK; 2 Department of Forensic Medicine and Toxicology, School of Medicine, National and Kapodistrian University of Athens, Athens, Greece; 3 Office of the Presidential Commissioner for Humanitarian Affairs, The Republic of Cyprus, Nicosia, Cyprus; 4 Laboratory of Forensic Genetics, 87198 The Cyprus Institute of Neurology and Genetics, Nicosia, Cyprus

**Keywords:** Forensic anthropology, DNA analysis, identification, gunshot trauma, Cyprus, colonialism

## Abstract

Forensic science has made some significant contributions to the investigation of human rights abuses related to armed conflicts, especially in the last 40 years. Some investigations are aimed at the collection of evidence in order to prosecute those responsible, while others are humanitarian in nature. This paper presents the multidisciplinary effort to recover and identify the remains of a 7-year-old child who was shot by British colonial forces in Cyprus in 1956. An investigation led to the discovery of the burial site, and archaeological methods were used to recover the remains. The anthropological examination provided information about the age of the child, as well as the nature of the skeletal trauma present. DNA results confirmed the identity of the victim, and the remains were released to the surviving family members for burial.

## Introduction

Forensic science has become widely known to the public through a variety of television programmes and depictions in film that goes back decades. However, when it comes to large-scale investigations, such as those dealing with disaster victim identification and human rights abuses, much less is known, despite their significance. Allegations of war crimes and crimes against humanity were first systematically investigated in Argentina in the mid-1980s, when forensic anthropologist Clyde Snow, with the help of local medical and archaeology students, examined gravesites containing the remains of victims of the military regime. This led to the foundation of the Argentine Forensic Anthropology Team in 1984.^1^ Other organisations were later formed in response to the increased need for human rights and humanitarian work, including Physicians for Human Rights and the Guatemalan Forensic Anthropology Foundation. The International Criminal Tribunals for the former Yugoslavia and Rwanda, International Criminal Court and Special Court for Sierra Leone all followed, as the need for evidence-based investigations was recognised.^2^ The International Commission of Missing Persons and the Forensic Unit of the International Committee of the Red Cross also made major contributions to this field, along with the most recent addition by the American Academy of Forensic Sciences, the Humanitarian and Human Rights Resource Center.^3^

Investigations concerned with human rights violations are multidisciplinary, as they require the information gathering and evaluation (various investigators) and location of graves and excavation (geophysicists and archaeologists). Once human remains have been exhumed, the post-mortem examinations and evidence management/recording take place (pathologists, anthropologists, odontologists, radiologists and geneticists), until the positive identification of the victims and repatriation of their remains. While some of these large-scale projects are forensic in nature, aiming to collect evidence for use in courts, others are purely humanitarian with the purpose of identifying remains and returning them to their loved ones to receive proper burial. Some projects combine the two approaches and are both forensic and humanitarian.^2,4^

The current study employs investigative, archaeological, anthropological and genetic methods to present a case from the colonial period of Cyprus.

## Case report

Cyprus is an island country in the eastern Mediterranean, composed mainly of two communities: Greek-Cypriots (82%) and Turkish-Cypriots (18%), according to the 1960 census when the country became independent.^5^ British presence in the country dates to 1878, when the Ottoman Empire ceded Cyprus to Great Britain. In 1925, the country became formally a colony of the British Empire. On 1 April 1955, the National Organisation of Cypriot Fighters, known as EOKA began its struggle against colonial rule.^6^ Over the next 4 years, hundreds of Cypriots and members of the British forces were killed. In some cases, captured EOKA members were hanged by the colonial authorities and buried on prison grounds in an attempt to prevent mass protests. In August 1960, Cyprus gained independence, becoming a member of the United Nations and Commonwealth. The newly founded state's peace was interrupted by inter-communal fighting in 1963 and 1964, resulting in many casualties from both sides. In 1974, following a coup in Cyprus by the military dictatorship ruling Greece at the time, Turkey invaded the island. The country was divided into the free south, which is internationally recognised as the legal Cypriot government, and the Turkish-occupied north. In 1999, the Republic of Cyprus government began investigating burials in areas under its control, establishing the Cyprus Exhumation and Identification Project. The aim was to exhume and identify for humanitarian reasons the remains of 1963–1964 victims, as well as the 1974 conflict, so that families of the Missing may learn the fate of their loved ones. Initial stages of the project were conducted with assistance from the non-governmental organisation Physicians for Human Rights.^7^

### Investigation

In 2021, a family submitted a request to the Presidential Commissioner for Humanitarian Affairs for the location, recovery and identification of the remains of their relative. He was shot by a British army officer on 14 March 1956, during a pupil protest against colonial rule. He was 7 years old.^8^ According to witness testimonies, the boy was shot in the head, and then buried hastily at a cemetery in the city of Larnaca, to prevent further demonstrations. Later, the burial site was altered and a cross bearing his name vanished. As a result, his family did not have a location to visit and exercise the funerary rites of their religion.

An investigation by the Office of the Presidential Commissioner identified a multiple family grave where the remains may have been located. An exhumation order was issued by the Larnaca district court, and a forensic team recovered the commingled remains of eight adults and one juvenile from the common grave. After a brief examination, the bones belonging to adults were returned to the grave, while the juvenile remains were taken to the Cyprus Exhumation and Identification Project laboratory for anthropological examination. Bone samples for DNA analysis were submitted to the Laboratory of Forensic Genetics (LabFoG) of the Cyprus Institute of Neurology and Genetics. Information on the family tree was obtained from a living paternal half-brother of the victim. Due to the lack of other close relatives in Cyprus, it was decided to exhume and take skeletal samples from the remains of the mother and a brother. Both exhumations were conducted by archaeologists from the Office of the Presidential Commissioner, following appropriate written consent from the living relatives. Femora from the exhumed mother and brother, as well as buccal swabs from living relatives, including a half-brother, were submitted to the LabFoG. A cranial vault fragment from the deceased juvenile was used to extract comparative genetic material.

### Anthropological and genetic examinations

**Figure 1.**
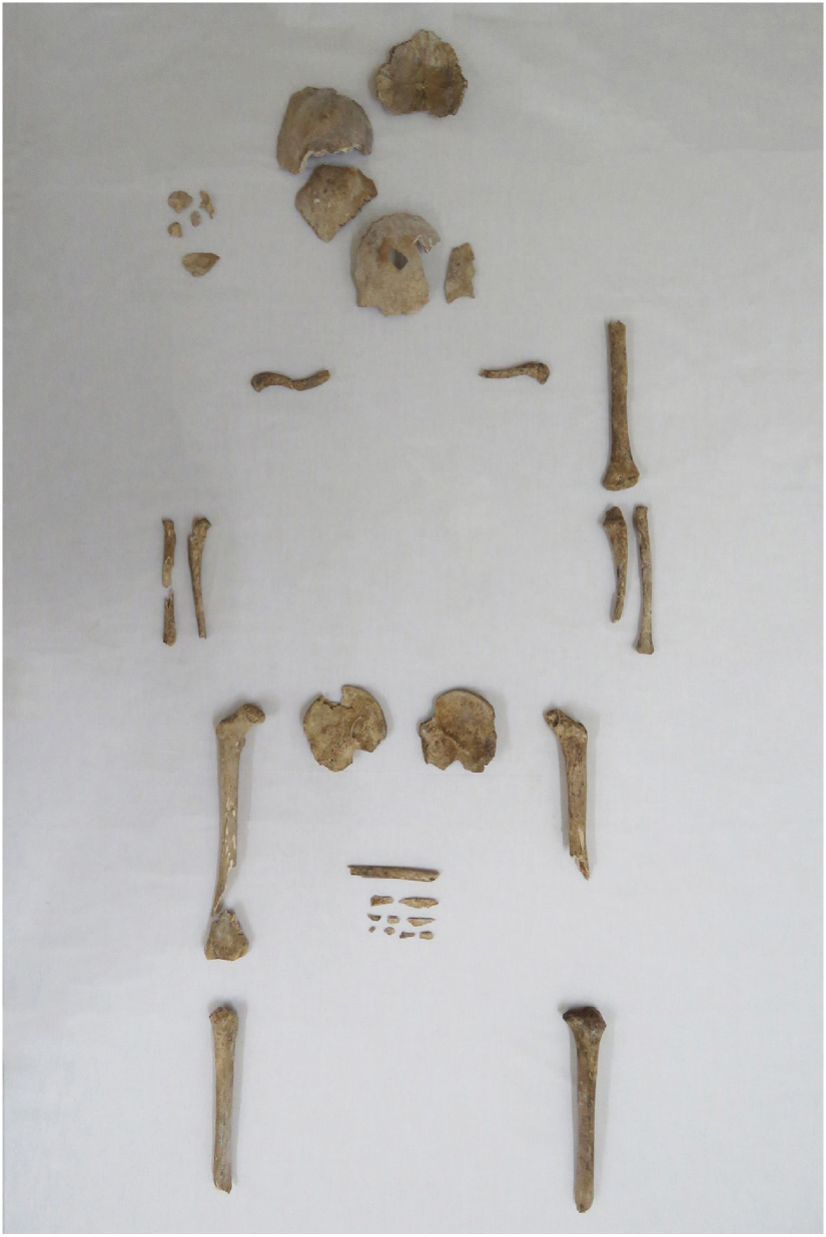
Overview of the remains identified as belonging to the 7-year-old victim in anatomical position.

**Figure 2.**
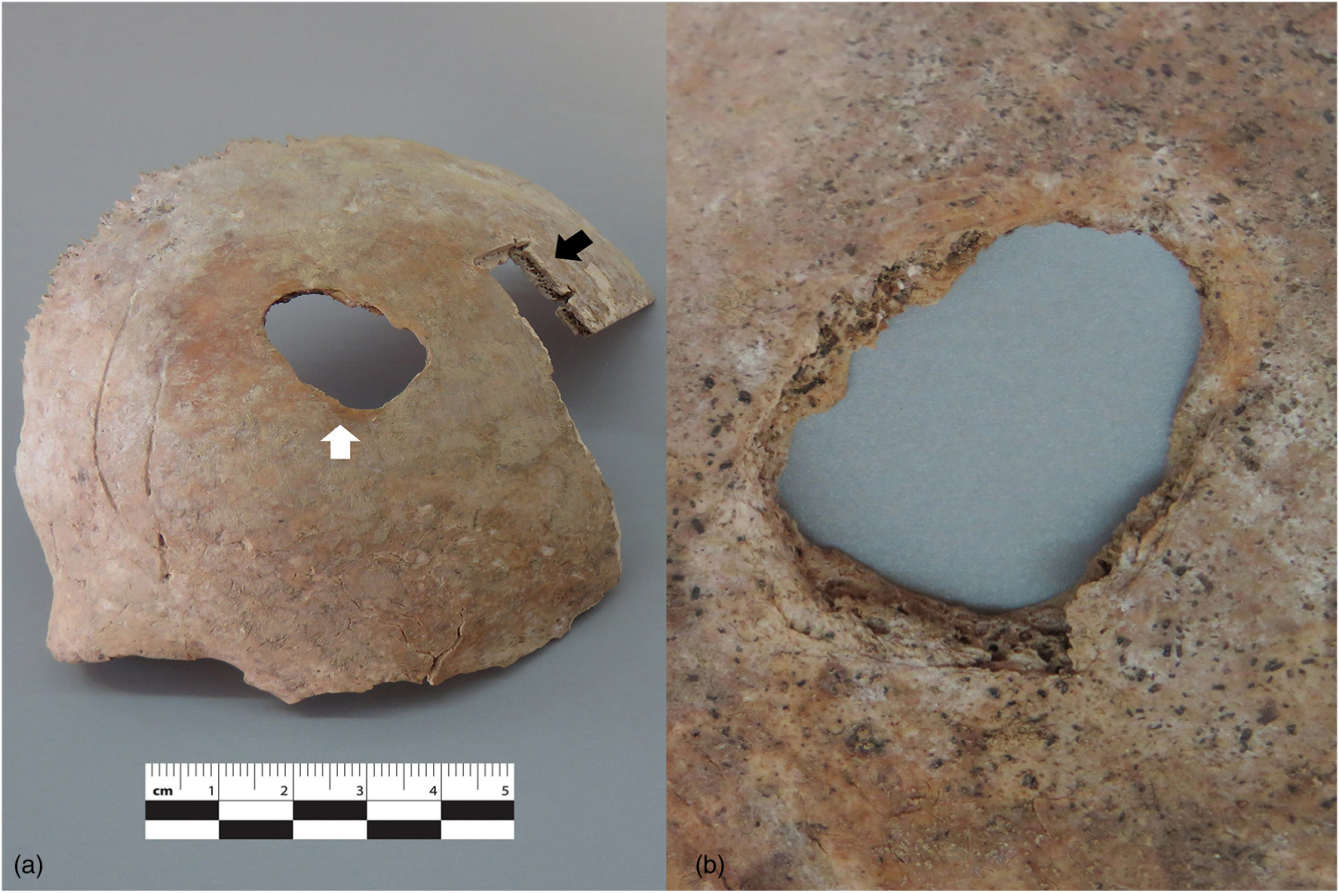
Frontal bone depicting projectile trauma. (a) Overview, showing the entrance wound *(white arrow)* and the area sampled for DNA analysis *(black arrow)*. (b) Close-up of the inner surface of the bone, demonstrating internal bevelling.

The anthropological examination indicated that the skeleton of the suspected victim was incomplete (Figure 1), and the bones were found to have cortical erosion, with cracking and fragmentation, taphonomic changes that are usually encountered in remains from a cemetery context in this part of the world.^9^ The cranium was represented by an almost complete frontal bone found in two fragments (>85%), an almost complete right parietal in two fragments (>90%), an almost complete occipital (>90%), as well as five cranial fragments measuring less than 2 × 3 cm in size. There was no mandible present. For the postcranial skeleton, none of the unfused long bone epiphyses was recovered and similarly, the spine, ribs, hand and foot bones were absent. The right clavicle was missing a small portion (<10%) of the sternal end, while the left clavicle was missing approximately the medial half (40%). The right humerus was not present, while the left was missing the head and a small portion of the proximal shaft (<10%). The right radius was fragmented at midshaft and almost complete, missing only a small portion (<15%) and the left radius was complete. The right ulna was missing a small portion of the distal part (<15%), while the left presented a similar level of completeness, also missing a small distal portion (<20%). The pelvic girdle was represented only by the two ilia that were almost complete and missing only small portions (<15%). The right femur was found in two fragments due to a post-mortem fracture at the distal end of the shaft and was missing a small portion (<15%). Only the proximal portion of the left femur was recovered and a large part of the distal part (<40%) was missing. The right tibia was missing a small portion of the proximal (<5%) and another (<15%) of the distal ends. The left tibia was missing only the distal part (<15%). In addition, a long bone shaft fragment measuring approximately 1 × 10 cm was recovered, along with three bone fragments that were not identified, which measured less than 4 × 1 cm. Six smaller unidentified fragments measuring less than 1 × 1 cm were also found. The sex of the remains could not be assessed by anthropological methods, as the individual had not reached skeletal maturity. The individual's age-at-death was estimated using long bone diaphyseal length. In this case, only the left radius was intact, with a maximum length of 14.8 cm corresponding to an age between 6 and 7 years.^10,11^ It should be noted that this estimate was based on the assumption that the individual was male, which was later confirmed by the DNA results. No antemortem trauma or pathologies were observed on the remains. In regards to perimortem trauma, a single projectile entry wound was present on the frontal bone, ∼50 mm superior of and medial to the right orbit (Figure 2). It measures ∼20 mm in a superior–inferior direction and 15 mm medio-laterally and is consistent with a tangential entry of a projectile. The direction of impact is confirmed by diagnostic characteristics: the outer cranial table has a sharp edge, while the inner presents bevelling.^12,13^ No radiating, or concentric fractures advancing from the impact site were present.^14^ No evidence of an exit wound could be located because a large part of the cranium was missing. A small portion of the frontal bone, located close to the site of trauma was submitted for DNA analysis (Figure 2).

Mitochondrial (HVI and HVII),^15^ Y-chromosome (Power-Plex-Y23)^16^ and autosomal STR (Powerplex-16 HS, PowerPlex-ESX17 Fast and MiniFiler)^17–19^ profiles were obtained from the remains and reported family members. The results provided a positive identification of the victim with a certainty of 99.999999844%.

## Conclusion

Through multidisciplinary collaboration by numerous individuals and agencies, the identified remains of the murdered child were recently laid to rest, 66 years after his death. Provided with his own, individual grave in the cemetery of Agios Georgios Kontou in Larnaca, his family was finally given the opportunity to have some closure. The present case demonstrates that the effects of colonialism can have long-lasting consequences that persist even today. In recent years, there is a growing trend to raise awareness about colonialism and this case serves as one example of how forensic science can shed light on the past, with the hope that such crimes will not be repeated in the future.
